# Empiric Piperacillin-Tazobactam *versus* Carbapenems in the Treatment of Bacteraemia Due to Extended-Spectrum Beta-Lactamase-Producing Enterobacteriaceae

**DOI:** 10.1371/journal.pone.0153696

**Published:** 2016-04-22

**Authors:** Tat Ming Ng, Wendy X. Khong, Patrick N. A. Harris, Partha P. De, Angela Chow, Paul A. Tambyah, David C. Lye

**Affiliations:** 1 Department of Pharmacy, Tan Tock Seng Hospital, Singapore, Singapore; 2 Division of Infectious Diseases, University Medicine Cluster, National University Hospital, Singapore, Singapore; 3 Yong Loo Lin School of Medicine, National University of Singapore, Singapore, Singapore; 4 University of Queensland, UQ Centre for Clinical Research, Brisbane, Queensland, Australia; 5 Department of Laboratory Medicine, Tan Tock Seng Hospital, Singapore, Singapore; 6 Department of Clinical Epidemiology, Institute of Infectious Diseases and Epidemiology, Tan Tock Seng Hospital, Singapore, Singapore; 7 Lee Kong Chian School of Medicine, Nanyang Technological University, Singapore, Singapore; 8 Department of Medicine, National University of Singapore, Singapore, Singapore; 9 Department of Infectious Diseases, Institute of Infectious Diseases and Epidemiology, Tan Tock Seng Hospital, Singapore, Singapore; University of Calgary, CANADA

## Abstract

Extended-spectrum beta-lactamase (ESBL)-producing Enterobacteriaceae are a common cause of bacteraemia in endemic countries and may be associated with high mortality; carbapenems are considered the drug of choice. Limited data suggest piperacillin-tazobactam could be equally effective. We aimed to compare 30-day mortality of patients treated empirically with piperacillin-tazobactam versus a carbapenem in a multi-centre retrospective cohort study in Singapore. Only patients with active empiric monotherapy with piperacillin-tazobactam or a carbapenem were included. A propensity score for empiric carbapenem therapy was derived and an adjusted multivariate analysis of mortality was conducted. A total of 394 patients had ESBL-*Escherichia*.*coli* and ESBL-*Klebsiella pneumoniae* bacteraemia of which 23.1% were community acquired cases. One hundred and fifty-one received initial active monotherapy comprising piperacillin-tazobactam (n = 94) or a carbapenem (n = 57). Patients who received carbapenems were less likely to have health-care associated risk factors and have an unknown source of bacteraemia, but were more likely to have a urinary source. Thirty-day mortality was comparable between those who received empiric piperacillin-tazobactam and a carbapenem (29 [30.9%] vs. 17 [29.8%]), P = 0.89). Those who received empiric piperacillin-tazobactam had a lower 30-day acquisition of multi-drug resistant and fungal infections (7 [7.4%] vs. 14 [24.6%]), P<0.01). After adjusting for confounders, use of empiric piperacillin-tazobactam was not associated with increased 30-day mortality (OR 1.00, 95% CI; 0.45–2.17). Empiric piperacillin-tazobactam was not associated with increased 30-day mortality and may result in fewer multi-drug resistant and fungal infections when compared with a carbapenem.

## Introduction

Extended-spectrum beta-lactamases (ESBL) are a subset of beta-lactamases that hydrolyse penicillins, cephalosporins and monobactams while cephamycins and carbapenems remain stable. Beta-lactam/beta-lactamase inhibitors have been shown to have variable activity against ESBL-producers [1]. TEM, SHV and CTX-M are the 3 main families of ESBLs with CTX-M becoming increasingly prevalent, particularly in *Escherichia coli* and *Klebsiella pneumoniae* [2]. Multiple genetic mechanisms were purported in the dissemination of CTX-M genetic determinants, providing the allotypic diversity allowing its rapid spread [3]. These mobile genetic elements frequently transmit resistance to classes of antimicrobial agents not hydrolysed by ESBL such as fluoroquinolones, aminoglycosides and trimethoprim/sulfamethoxazole [4].

There is an increasing prevalence of ESBL-producing Enterobacteriaceae in the United States, Europe and Asia-Pacific region [5–7]. ESBL-producing Enterobacteriaceae are most commonly detected in healthcare settings but their prevalence has also been increasing in the community [8]. At our institution in Singapore, 12.4% of patients admitted via the emergency department were found to be colonised [9] across all public institutions in Singapore, 17.5% of *E*.*coli* and 35.9% of *K*. *pneumoniae* were ESBL-positive [10]. These organisms were associated with high mortality [1, 11, 12]. Based on observational data, carbapenems have been the drug of choice for treating severe infections caused by ESBL producers [12]. However, there is emerging evidence that piperacillin-tazobactam or cefepime may be an effective alternative antibiotic and may reduce the selection pressure for carbapenem resistance [13–16].

Carbapenem-resistant Enterobacteriaceae have spread globally since first reported in 2010 [17–19]. Therapeutic options are limited, increasing the complexity of managing these infections [20]. Loss of outer-membrane porins leading to carbapenem resistance in CTX-M producing strains was also reported [18]. This type of resistance may be reduced in the absence of selection pressure; it is possible that carbapenem-resistant mutants can be selected during carbapenem therapy [3, 21]. Antimicrobial stewardship plays an important part in controlling multidrug-resistant organisms and reducing carbapenem usage may help contain the spread of carbapenem resistance. Therefore, it is important to identify effective alternative antibiotics that can be used empirically in an environment with high ESBL Enterobacteriaceae endemicity.

In this study, we aimed to compare 30-day mortality of patients with ESBL-producing *E*. *coli* and *K*. *pneumoniae* bacteraemia treated empirically with piperacillin-tazobactam versus a carbapenem in a multi-centre study in Singapore.

## Methods

### Study design and patients

A retrospective cohort study was conducted at 2 university teaching hospitals in Singapore. Tan Tock Seng Hospital (TTSH) has a capacity of 1500 beds while the National University Hospital (NUH) has 1000 beds. Ethics approval was obtained from National Healthcare Group domain specific review boards (Approval number 2013/00083). Patient information was anonymized and de-identified prior to data collection and analysis. Patients with ESBL-producing *E*. *coli* and *K*. *pneumoniae* bacteraemia from August 2011 to May 2013 at TTSH and May 2012 to May 2013 at NUH were identified from their respective electronic microbiology databases. For patients with multiple episodes of ESBL-producing *E*. *coli* or *K*. *pneumoniae* bacteraemia, only the first episode was included. Patients were excluded if they had polymicrobial bacteraemia, or did not receive at least 48 hours of empirical or definitive antimicrobial therapy. Subjects were followed up until discharge or death, whichever was earlier. Data were collected from the electronic medical records. Data collected included patient demographics, microbiology data, empiric and definitive antibiotic therapy, source of bacteraemia, Charlson’s co-morbidity index, Pitt bacteraemia score, and clinical outcomes. Source of bacteraemia was defined using published criteria [22].

### Definitions

Active empiric antibiotic therapy referred to the use of antibiotics before susceptibility was known, started within 24 hours of blood culture collection with subsequent matching *in vitro* susceptibility and continued for at least 48 hours. Oxyimino-beta-lactams such as cefuroxime, ceftriaxone, ceftazidime, and aztreonam were considered inactive even if they demonstrated *in vitro* susceptibility. Definitive antibiotic therapy referred to the use of antibiotic after *in vitro* susceptibility was known. Nosocomial bacteraemia was defined by a positive blood culture obtained after 48 hours of admission [22]. Healthcare-associated bacteraemia was defined as a positive blood culture obtained within 48 hours of hospital admission and if the patient fulfilled any healthcare associated risk factors [23]. Patients with no nosocomial or healthcare-associated risk factors were defined as community-acquired bacteraemia. Doses of antibiotics used are according to the respective hospital guidelines and usual doses are as follows: piperacillin-tazobactam 4.5 g every infused over 30 min every 6 hours or 4.5g every infused over 3 hours every 8 hours, ertapenem 1 g every 24h hours, imipenem 500 mg every 6 hours and meropenem 1 g every 8 hours. All doses were adjusted for renal function accordingly.

### Laboratory methods

The study included patients with *E*. *coli* or *K*. *pneumoniae* isolated from at least one positive blood culture, with resistance to third-generation cephalosporins and demonstrated susceptibility to both piperacillin-tazobactam and carbapenems. There were minor differences in the methods used in each hospital laboratory. In the TTSH laboratory, blood cultures were incubated using the Bactec 9240 system (Becton Dickinson, Maryland, USA) with susceptibility testing performed using disk diffusion and Clinical and Laboratory Standards Institute (CLSI) criteria (Performance Standards for Antimicrobial Disk Susceptibility Tests; Approved standard- Eleventh Edition 2012, CLSI). The NUH laboratory used the BacT/Alert blood culture system (BioMerieux, France) and automated microbroth dilution (Vitek 2, BioMerieux) for susceptibility testing, according to EUCAST interpretative standards (www.eucast.org).

### Outcomes assessment

Thirty-day mortality was defined as death within 30 days of an ESBL-producing *E*. *coli* or *K*. *pneumoniae* bacteraemia. Multi-drug resistant organisms were defined as positive clinical cultures of methicillin-resistant *S*. *aureus*, vancomycin-resistant e*nterococci*, carbapenem and/or piperacillin-tazobactam resistant gram-negative bacteria, other organisms resistant to more than 3 classes of antibiotics and fungal infections within 30 days of index bacteraemia were noted. Relapsed bacteraemia was defined as return of the same organism as the index bacteraemia in positive blood cultures more than 72 hours after but within 30 days of the index episode.

### Statistical analysis

Categorical variables were compared using the Chi-square or Fisher’s exact tests where applicable while continuous variables were compared using the Mann–Whitney U-test. A P-value of <0.05 was considered significant. We constructed a multivariable logistic regression model to estimate a propensity score for each patient’s probability of receiving an empiric carbapenem [24]. Covariates were identified by comparing the group receiving empiric piperacillin-tazobactam with an empiric carbapenem. Covariates that were significantly different between both groups on univariate analysis were entered into the multivariate model. The Hosmer and Lemenshow test was used to assess the fit of the model. Univariate analysis was used to identify variables associated with 30-day mortality. The propensity score of receiving carbapenem, Pitt bacteraemia score, Charlson’s co-morbidity index, empiric piperacillin-tazobactam, and variables with P<0.1 from univariate analysis were included in a multivariable logistic model for risk factors for 30-day mortality. Confounders of other clinical outcomes where relevant were analysed and adjusted in a similar fashion, and included in the propensity score of receiving a carbapenem, the Pitt bacteraemia score, Charlson’s co-morbidity index and variables with P<0.1 from univariate analysis. All analyses were performed using SPSS version 20 (IBM Corp, Armonk, NY, USA).

## Results

A total of 394 patients had ESBL-*E*. *coli* and ESBL-*K*. *pneumoniae* bacteraemia at the two hospitals during the respective study periods. Twenty-three percent were community acquired. One hundred and fifty-one patients received active empiric monotherapy comprised of either piperacillin-tazobactam (n = 94) or a carbapenem (n = 57). Patients who received a carbapenem were less likely to have health-care associated risk factors (22 [38.6%] vs. 56 [59.6%]) and have an unknown source of bacteremia (1 [1.8%] vs. 14 [14.9%]). They were more likely to have a urinary source (40 [70.2%] vs. 49 [52.1%]) (Table 1). These significant factors were used to construct an individual patient propensity score of receiving an empiric carbapenem. The Hosmer and Lemenshow statistics of the multivariable logistic regression model was P = 0.99.

**Table 1:** Characteristics of patients with ESBL-producing *E*. *coli* and *K*. *pneumoniae* bacteremia, according to treatment type.

Characteristic	Empiric active piperacillin-tazobactam (N = 94)	Empiric carbapenem (N = 57)	P value
Age, median (IQR)	79 (70–85)	78 (69–84)	0.67
Male sex	45 (47.9)	31 (54.4)	0.44
Nosocomial onset	33 (35.1)	27 (47.4)	0.14
Healthcare-associated onset	56 (59.6)	22 (38.6)	0.01
Community acquired	5 (5.3)	8 (14.0)	0.08
*E*.*coli* bacteraemia	62 (66.0)	39 (68.4)	0.76
ICU admission	5 (5.3)	8 (14.0)	0.08
Charlson’s comorbidity index, median (IQR)	6 (5–7)	6 (4–8)	0.44
Pitt bacteraemia score (IQR)	1 (0–3)	1 (0–3)	0.91
Source of bacteremia			
Urinary	49 (52.1)	40 (70.2)	0.03
Unknown	14 (14.9)	1 (1.8)	0.01
Hepatobiliary	11 (11.7)	3 (5.3)	0.19
Respiratory	9 (9.6)	4 (7.0)	0.77
Intra-abdominal	4 (4.3)	4 (7.0)	0.48
Intravascular catheter	3 (3.2)	3 (5.3)	0.67
Others	2 (3.2)	1 (2.6)	1.00
30-day mortality	29 (30.9)	17 (29.8)	0.89
Length of stay after bacteraemia onset, median (IQR)	18 (10–30)	16 (8–24)	0.15
30-day CDAD acquisition	2 (2.1)	0 (0.0)	0.54
30-day acquisition of multidrug resistant bacterial and fungal infections	7 (7.4)	14 (24.6)	<0.01
30-day relapsed bacteraemia^a^	2/63 (3.2)	6/38 (15.8)	0.05

Data are no. of patients (%), unless otherwise indicated

^a^Denominator represents the number of patients with repeated blood cultures within 30 days and the numerator are the numbers with relapsed bacteraemia.

CDAD: *Clostridium difficile* associated diarrhoea

**Fig 1:**
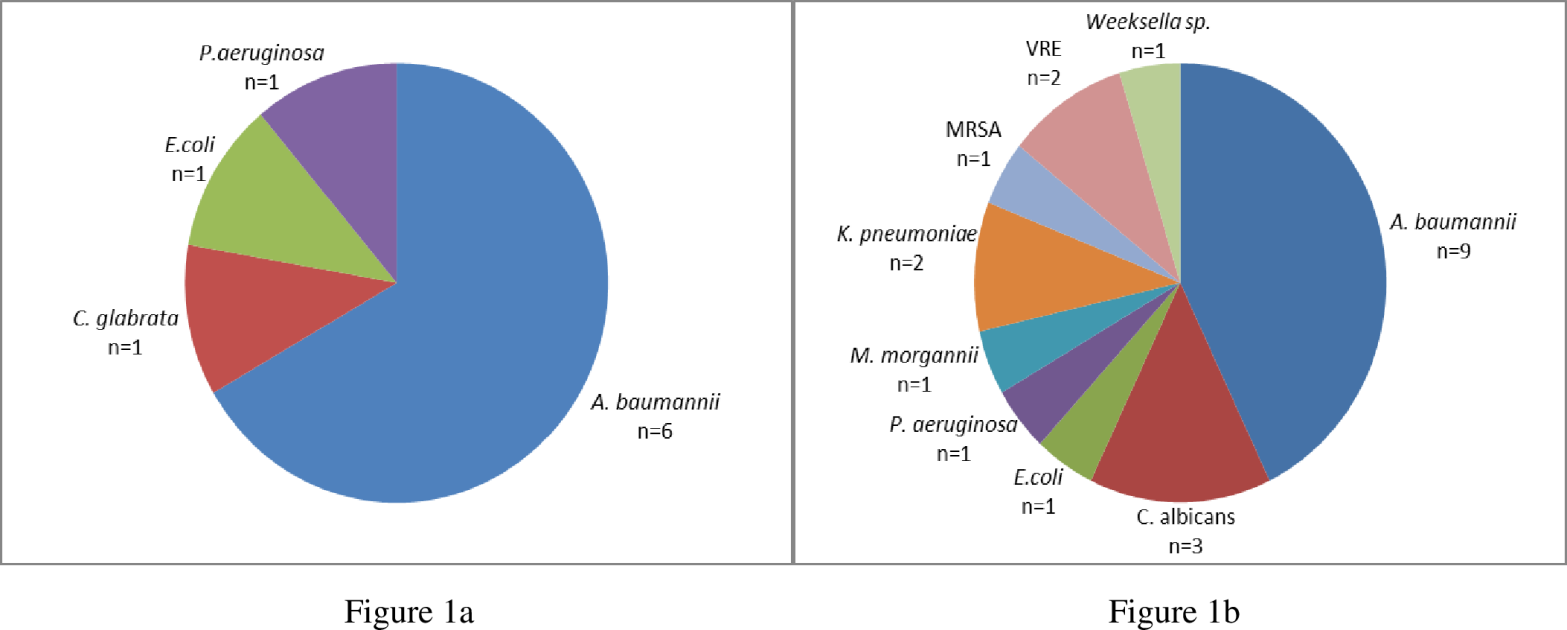
Types of multi-drug resistant bacterial and fungal infections acquired by patients who received empiric piperacillin-tazobactam and carbapenems^a^. ^a^Patients with multiple multi-drug resistant organisms and fungal infections are only counted once. (**a):** Types of multi-drug resistant bacterial and fungal infections in patients who received empiric piperacillin-tazobactam. No. of patients 7/94, (7.4%). (**b):** Type of multi-drug resistant bacterial and fungal infections in patients who received empiric carbapenem. No. of patients 14/57, (24.6%).

Thirty-day mortality was comparable between those who received empiric piperacillin-tazobactam and carbapenem (29 [30.9%] vs. 17 [29.8%]), P = 0.89). After adjusting for confounders, use of empiric piperacillin-tazobactam was not associated with increased 30-day mortality (OR 1.00, 95% CI; 0.45–2.17) (Table 2). Those who received empiric piperacillin-tazobactam had a lower 30-day acquisition of multi-drug resistant bacterial and fungal infections (7 [7.4%] vs. 14 [24.6%]), P<0.01) (Fig 1). Multivariable analysis showed that an empiric carbapenem was the only significant risk factor for acquisition of multi-drug resistant bacterial and fungal infections (OR 3.32, 95% CI; 1.12–9.87) (Table 3).

**Table 2:** A propensity score for receiving carbapenem adjusted, multivariable analysis of 30-day mortality for 151 patients with ESBL-producing *E*. *coli* and *K*. *pneumoniae* bacteraemia, receiving either empiric piperacllin-tazobactam or carbapenem.

Variable	Univariate analysis		Multivariable analysis	
	OR, (95% CI)	p	AOR, (95% CI)	p
Pitt bacteraemia score	1.11 (0.90–1.36)	0.32	1.20 (0.98–1.48)	0.08
Charlson’s comorbidity index	0.97 (0.84–1.11)	0.61	0.94 (0.81–1.09)	0.40
Respiratory source	2.96 (0.94–9.37)	0.07	2.81 (0.87–9.05)	0.08
Hepatobiliary source	0.16 (0.02–1.24)	0.08	0.18 (0.02–1.48)	0.11
Unknown source	2.95 (1.00–8.69)	0.05	1.51 (0.33–6.92)	0.60
Empiric piperacillin-tazobactam	1.05 (0.51–2.15)	0.89	0.99 (0.45–2.17)	0.99

Abbreviations: OR, Odds ratio; AOR, propensity score adjusted odds ratio; CI, confidence interval; Model fit: Hosmer and Lemenshow P = 0.99

The propensity score for receiving empiric carbapenems were included in the multivariable logistic regression model; OR 1.20, 95% CI; 0.06–24.44)

**Table 3:** A propensity score for receiving carbapenem adjusted, multivariable analysis of 30-day acquisition of multi-drug resistant bacterial and fungal infections for 151 patients with ESBL-producing *E*. *coli* and *K*. *pneumoniae* bacteraemia, receiving either empiric piperacllin-tazobactam or carbapenem.

Variable	Univariate analysis		Multivariable analysis	
	OR, (95% CI)	p	AOR, (95% CI)	p
Pitt bacteraemia score	1.28 (1.00–1.64)	0.05	1.39 (0.98–1.97)	0.06
Charlson’s comorbidity index	0.93 (0.77–1.12)	0.42	0.89 (0.72–1.09)	0.26
ICU admission	3.16 (0.88–11.41)	0.08	0.97 (0.12–7.77)	0.97
Nosocomial acquired bacteraemia	3.65 (1.38–9.69)	<0.01	2.15 (0.38–12.01)	0.38
Healthcare-associated bacteraemia	0.24 (0.08–0.71)	<0.01	0.70 (0.08–5.96)	0.75
Intra-abdominal source	4.17 (0.92–18.94)	0.07	3.74 (0.65–21.52)	0.14
Empiric carbapenem	4.05 (1.52–10.76)	<0.01	3.32 (1.12–9.87)	0.03

Abbreviations: OR, Odds ratio; AOR, propensity score adjusted odds ratio; CI, confidence interval; ICU, Intensive Care Unit

Model fit: Hosmer and Lemenshow P = 0.89

Among those patients with repeat blood cultures within 30 days, there were more patients in the empiric carbapenem group who experienced relapsed bacteraemia compared with those that received empiric piperacillin-tazobactam (6/38 [15.8%] vs. 2/63 [3.2%]), P = 0.05). All patients who relapsed were those who had ESBL-*E*. *coli* initially. However, a multivariable analysis taking into account other confounding variables showed that empiric carbapenem was not associated with relapsed bacteraemia (OR 6.84, 95% CI; 0.81–58.3, P = 0.08) (Table 4).

**Table 4:** A propensity score for receiving carbapenem adjusted, multivariable analysis of 30-day relapse bacteraemia for 101 patients with ESBL-producing *E*. *coli* and *K*. *pneumoniae* bacteraemia, receiving either empiric piperacllin-tazobactam or carbapenem.

Variable	Univariate analysis		Multivariable analysis	
	OR, (95% CI)	p	AOR, (95% CI)	p
Male	6.29 (0.74–53.13)	0.09	5.25 (0.53–51.31)	0.15
Charlson’s comorbidity index	0.80 (0.59–1.07)	0.13	0.89 (0.63–1.25)	0.49
Pitt bacteraemia	0.78 (0.44–1.38)	0.40	1.09 (0.54–2.21)	0.82
Community acquired bacteraemia	4.83 (0.80–29.28)	0.09	5.51 (0.59–51.55)	0.14
Empiric carbapenem	5.72 (1.09–29.97)	0.04	6.84 (0.80–58.33)	0.08
Neutropenic sepsis	13.14 (0.74–233.28)	0.08	10.76 (0.27–426.50)	0.21

Abbreviations: OR, Odds ratio; AOR, propensity score adjusted odds ratio; CI, confidence interval

Model fit: Hosmer and Lemenshow P = 0.97

A total of 149 of 151 (98.5%) patients received active definitive therapy. One patient died before blood cultures results were available and one received in-active ciprofloxacin. Among those who received definitive active therapy, 10 (6.7%) patients received combination definitive therapy. Among the 56 patients who received an empiric carbapenem, 5 patients received combination definitive therapy of piperacillin-tazobactam and a carbapenem (n = 1), a carbapenem and co-tromixazole (n = 2), piperacillin-tazobactam and amikacin (n = 1) and a carbapenem plus ciprofloxacin (n = 1) respectively. The remaining 51 patients received definitive monotherapy consisting of a carbapenem (n = 49), amikacin (n = 1) and ciprofloxacin (n = 1). Among the 93 patients who received empiric piperacillin-tazobactam, 5 patients received definitive combination therapy of piperacillin-tazobactam and a carbapenem (n = 2), piperacillin-tazobactam and ciprofloxacin (n = 1), piperacillin-tazaobctam and a carbapenem with aminoglycoside (n = 2) respectively. The remaining 88 patients received definitive monotherapy consisting of a carbapenem (n = 79) and piperacillin-tazobactam (n = 9).

One hundred and one (66.9%) patients had ESBL-*E*. *coli* and 50 (33.1%) had ESBL-*K*. *pneumoniae* bacteraemia. There were more community acquired bacteraemia (13 [12.9%] vs. 0 [0.0%]), P<0.01) and healthcare-associated onset of bacteraemia (60 [59.4%] vs. 18 [36.0%], P<0.01) among patients with ESBL-*E*. *coli* bacteraemia. ESBL-*K*. *pneumoniae* bacteraemia were more often nosocomial acquired (32 [64.0%] vs. 28 [27.7%], P<0.01). Other baseline characteristics were comparable. Out of those patients with repeat blood cultures within 30 days, 8 (12.3%) patients with ESBL-*E*. *coli* bacteraemia experienced a relapse while there was no relapse in those with ESBL-*K*. *pneumoniae* bacteraemia.

## Discussion

Our study showed that there was a significant burden of community-acquired ESBL-producing *E*. *coli and K*. *pneumoniae* bacteraemia in Singapore. However, even in this setting of high endemicity, this multi-centre study found no difference in 30-day mortality and length of hospitalisation between patients treated with piperacillin-tazobactam and a carbapenem. However, patients who received empiric carbapenems had an increased risk of multi-drug resistant bacterial and fungal infections. There was a non-significant trend towards increased relapse bacteraemia in those who received empiric carbapenems.

Patients who received piperacillin-tazobactam more often had bacteraemia that were healthcare-associated compared to those who received a carbapenem. Majority of the bacteraemia were from TTSH (86.1%). In TTSH, antibiotic guidelines recommend piperacillin-tazobactam for healthcare-associated pneumonia and it is often used empirically for infections that are healthcare-associated. Healthcare-associated risk factors considered were prior hospital admissions in the last 90 days, nursing home residence, haemodialysis and intravenous chemotherapy. However, this practice of recommending empiric piperacillin-tazobcatam for healthcare-associated infections was not routinely practised in NUH.

Carbapenems were suggested as the drug of choice for treating severe infections. In a previous prospective study of 85 episodes of ESBL-producing *K*. *pneumoniae* bacteraemia, carbapenem monotherapy was associated with lower 14-day mortality [12]. These early cohort studies which identified that carbapenems were associated with lower mortality had few subjects treated with beta-lactam/beta-lactamase inhibitors [12, 25]. In a cohort of 79 patients with *E*. *coli* and *K*. *pneumoniae* bacteraemia mainly from urinary source, patients treated empirically with a beta-lactam/beta-lactamase inhibitor had a higher but not statistically significant mortality compared to those who received other types of antibiotics (6 [38%] vs. 10 [18%], P = 0.06). The reverse was observed when comparing between patients who received carbapenems compared to those who did not (0 [0%] vs. 16 [30%], P = 0.09) [26]. A similar cohort of 79 patients with non-urinary source *E*. *coli* and *K*. *pneumoniae* bacteraemia who received either carbapenem or piperacillin-tazobactam had increased 90-day mortality in patients who received piperacillin-tazobactam (OR 7.9, 95% CI; 1.2–53). However, there was no significant difference when the more widely reported 30-day mortality was compared between both groups [27]. In a cohort of 331 ESBL bacteraemias who received definitive carbapenem therapy, after adjusting for confounders, there was a 1.92 times increased risk of death by day 14 for patients receiving empiric piperacillin-tazobactam compared with patients receiving empiric carbapenems [28]. The majority of the bacteraemias were of intra-abdominal origin and associated with central lines. It is possible that piperacillin-tazobactam may not be comparable to carbapenems for bacteraemia from non-urinary sources. However, only 39% received the 4.5g every 6 hourly dose, while 61% received doses of 3.375 g every 6 hours and no patient received extended infusions. We were not able to track the exact dosing regimens used in our retrospective cohort. It has been suggested that it is reasonable to treat ESBL Enterobactericeae with MIC ≤ 16 mg/l and with at least 3.375g 8 hour with 4 hour extended infusion [15]. Higher piperacillin-tazobactam minimum inhibitory concentration (MIC) (8–16 mg/L) was associated with poorer outcomes and extended infusions were suggested to overcome these issues [14,15]. More data are needed to elucidate the optimal dosing of piperacillin-tazobactam against ESBL organisms. In addition, it is important to recognise the “inoculum effect” which can diminish the effect of piperacillin-tazobactam [29]. However, there are limited data to describe the clinical impact of this phenomenon.

Our study results support the hypothesis that piperacillin-tazobactam can be comparable to carbapenems in ESBL bacteremia. A post-hoc analysis of patients with ESBL-producing *E*. *coli* bacteraemia, mainly from biliary and urinary sources, did not show a significant difference in 30-day mortality between patients treated with a carbapenem and beta-lactam/beta-lactamase inhibitors both empirically and definitively [14]. Further investigations by Kang *et al*. support these findings [30].

Early appropriate empiric therapy is believed to be critical in reducing mortality from bacteraemia [31]. As the majority of our cohort received definitive carbapenem therapy, any observed outcome differences could be attributed to the effects of empiric carbapenem and piperacillin-tazobactam. Our results suggested that piperacillin-tazobactam can be ***as*** effective as a carbapenem in the empiric treatment of sepsis with likely ESBL bacteraemia. In addition, empiric carbapenem was associated with a higher risk of acquisition of multi-drug resistant bacterial and fungal infections. With the emergence of carbapenemase-producing Enterobacteriaceae and the consequent need for an alternative to carbapenem antibiotics, our results support results from other studies [14,25] in reducing carbapenem usage as a safe and practical approach in antimicrobial stewardship [21].

Our study has several limitations. First of all, this study was a retrospective analysis and we could only control for confounders that we collected. We were unable to ascertain if all patients received extended infusions. We attempted to address differences in baseline characteristics of patients who received active empiric piperacillin-tazobactam and carbapenem by modelling the propensity for empiric carbapenem and adjusting for it in the multivariate analysis. Since the majority of bacteraemias were mainly from a urinary source, results from our study may not be generalizable to patients with other sources of bacteraemia. We did not analyse the impact of definitive therapy as most patients eventually received carbapenems. Lastly, routine testing of MIC of all antibiotics for treatment of ESBL infections was not performed in all cases.

## Conclusions

In conclusion, the use of empiric piperacillin-tazobactam in the treatment of ESBL-producing *E*. *coli* or *K*. *pneumoniae* bacteraemia in a cohort with mainly urinary tract infections was not associated with higher 30-day mortality compared with empiric carbapenems. Randomised controlled trials comparing carbapenems and “optimally dosed” piperacillin-tazobactam are needed and are underway [32].
